# Evaluation of the Environmental Impact and Efficiency of N-Doping Strategies in the Synthesis of Carbon Dots

**DOI:** 10.3390/ma13030504

**Published:** 2020-01-21

**Authors:** Suzanne Christé, Joaquim C.G. Esteves da Silva, Luís Pinto da Silva

**Affiliations:** 1 Chemistry Research Unit (CIQUP), Faculty of Sciences of University of Porto, R. Campo Alegre 697, 4169-007 Porto, Portugal; christe.suzanne@gmail.com (S.C.); jcsilva@fc.up.pt (J.C.G.E.d.S.); 2 LACOMEPHI, GreenUPorto, Department of Geosciences, Environment and Territorial Planning, Faculty of Sciences of University of Porto, R. Campo Alegre 697, 4169-007 Porto, Portugal

**Keywords:** carbon dots, photoluminescence, nitrogen doping, life cycle assessment, green chemistry, engineered nanomaterials

## Abstract

The efficiency and associated environmental impacts of different N-doping strategies of carbon dots (CDs) were evaluated. More specifically, N-doped CDs were prepared from citric acid via two main synthesis routes: Microwave-assisted hydrothermal treatment with addition of N-containing small organic molecules (urea and ethylenediamine (EDA)); and microwave-assisted solvothermal treatment in N-containing organic solvents (*n*,*n*-dimethylformamide (DMF), acetonitrile and pyridine). These syntheses produced CDs with similar blue emission. However, XPS analysis revealed that CDs synthesized via both hydrothermal routes presented a better N-doping efficiency (~15 at.%) than all three solvothermal-based strategies (0.6–7 at.%). However, from the former two hydrothermal strategies, only the one involving EDA as a nitrogen-source provided a non-negligible synthesis yield, which indicates that this should be the preferred strategy. This conclusion was supported by a subsequent life cycle assessment (LCA) study, which revealed that this strategy is clearly the most sustainable one from all five studied synthesis routes.

## 1. Introduction

Carbon dots (CDs) are carbon-based nanoparticles (NPs) with a core that is thought to be composed of sp^2^ carbon connected by sp^3^ carbon atoms in between [1,2,3]. On their surface can be found various types of functional groups (such as carboxylic acids, alcohols, and amines), which identity depends on the type of precursor used and synthesis route employed [4]. Interestingly, CDs possess several attractive features, such as good physical-chemical and photochemical stability, good water solubility, high photoluminescence, biocompatibility, broadband absorption, and low toxicity [5,6]. Furthermore, CDs can be produced through green and simple methods of synthesis [7], using even waste biomass as a precursor [8,9]. Given this, it is not surprising that CDs have attracted significant attention from the research community, and that they have been gaining several practical applications, such as in sensing [10,11,12], bioimaging [13], fabrication of light emission devices [14,15], and in photocatalysis [2].

CDs can be obtained via two main synthetic routes: top-down and bottom-up [1,2,3]. The first approach is based on the breakdown of large graphite materials in smaller carbon-based materials [16]. However, bottom-up methods, such as microwave-assisted processes [17,18,19,20], appear to be the most widely used for the preparation of CDs, which consist of the synthesis of CDs from smaller carbon precursors (small organic molecules) [21,22]. Among these, citric acid (CA) is one of the most used [17,23,24,25,26].

It should be noted that addition of additional precursors containing appropriate heteroatoms (such as N, S, P, and B) can lead to heteroatom-doping of CDs [3]. Nitrogen-doping (N-doping) is arguably the most often used heteroatom-doping, as the nitrogen and carbon atoms have comparable size. Further, nitrogen offers five valence electrons for bonding with carbon atoms [3]. More importantly, N-doping generally enhances the fluorescence quantum yield (QY_FL_) of the CDs [27,28,29], which has made N-doping one of the main strategies for enhancing the photoluminescence of CDs [3,30,31]. N-doping is generally achieved by adding a nitrogen-rich small organic molecule as a nitrogen precursor to the carbon precursor in hydrothermal-based bottom-up synthetic routes, with typical nitrogen precursors being molecules such as urea [17,26] and EDA [32,33].

However, and despite significant research, the effect of N-doping strategies on the QY_FL_ is still not fully understood. Given this, it is not easy to predict the efficiency exerted by using different nitrogen precursors on the QY_FL_ of the resulting CDs. This makes difficult the rational development of CDs with high QY_FL_, which is a feature required for most (if not all) current applications of these NPs [1,2,3,10,11,12,13,14,15]. In fact, it has not been fully assessed whether using either a nitrogen-rich solvent in a solvothermal route or a nitrogen-rich small organic molecule in a hydrothermal route would offer a greater potential. Another glaring lack of knowledge is the absence of data regarding the potential environmental impacts generated by different N-doping strategies. This is quite problematic since the production of nanomaterials has been found to be orders of magnitude more energy- and material-consuming than that of fine chemicals and pharmaceuticals [34], which identified it an environmental concern [35]. In fact, some studies have shown that the energy and chemicals used during the synthesis of nanomaterials contribute a significant share of the environmental impacts generated during their life cycle [36,37,38,39]. Thus, assessing the efficiency and sustainability of different N-doping strategies should be a priority in the study and development of CDs.

Herein, the aim of this work was to assess the efficiency and sustainability of different N-doping strategies employed during the synthesis. Namely, CDs prepared from CA (a typical carbon precursor [17,23,24,25,26]) were N-doped via two different strategies: (1) Microwave-assisted hydrothermal synthesis with addition of N-containing small organic molecules (urea or EDA), which are typically used as nitrogen precursors [17,26,32,33]; (2) microwave-assisted solvothermal synthesis in an N-containing solvent (DMF, acetonitrile or pyridine). The first step of this study was the characterization of the obtained CDs by X-ray photoelectron spectroscopy (XPS), UV–Visible (UV-Vis) spectroscopy and fluorescence spectroscopy, and by dynamic light scattering (DLS), which allowed us to determine the efficiency of N-doping and its effects on the photoluminescence of the CDs. The sustainability of each N-doping route was determined by performing a life cycle assessment (LCA) study of each synthesis. LCA is the most appropriate tool to address this issue, as it aims to quantify the environmental impacts of a given system during its life cycle [40,41]. Moreover, LCA has already been used in the environmental evaluation of different nanomaterials, such as silver NPs [36], copper NPs [37], carbon nanotubes [38], and magnetic NPs [39].

## 2. Materials and Methods 

The experimental section is divided into six sub-sections, which are relative to: Synthesis procedure for the fabrication of carbon dots (2.1); details regarding the characterization methods used (2.2); details regarding the method and calculation of fluorescence quantum yield QY_FL_ (2.3); scope and system boundaries for life cycle assessment (2.4); life cycle inventory data (2.5); and details relative to environmental impact assessment (2.6).

### 2.1. Synthesis of Carbon Dots

**Scheme 1:**
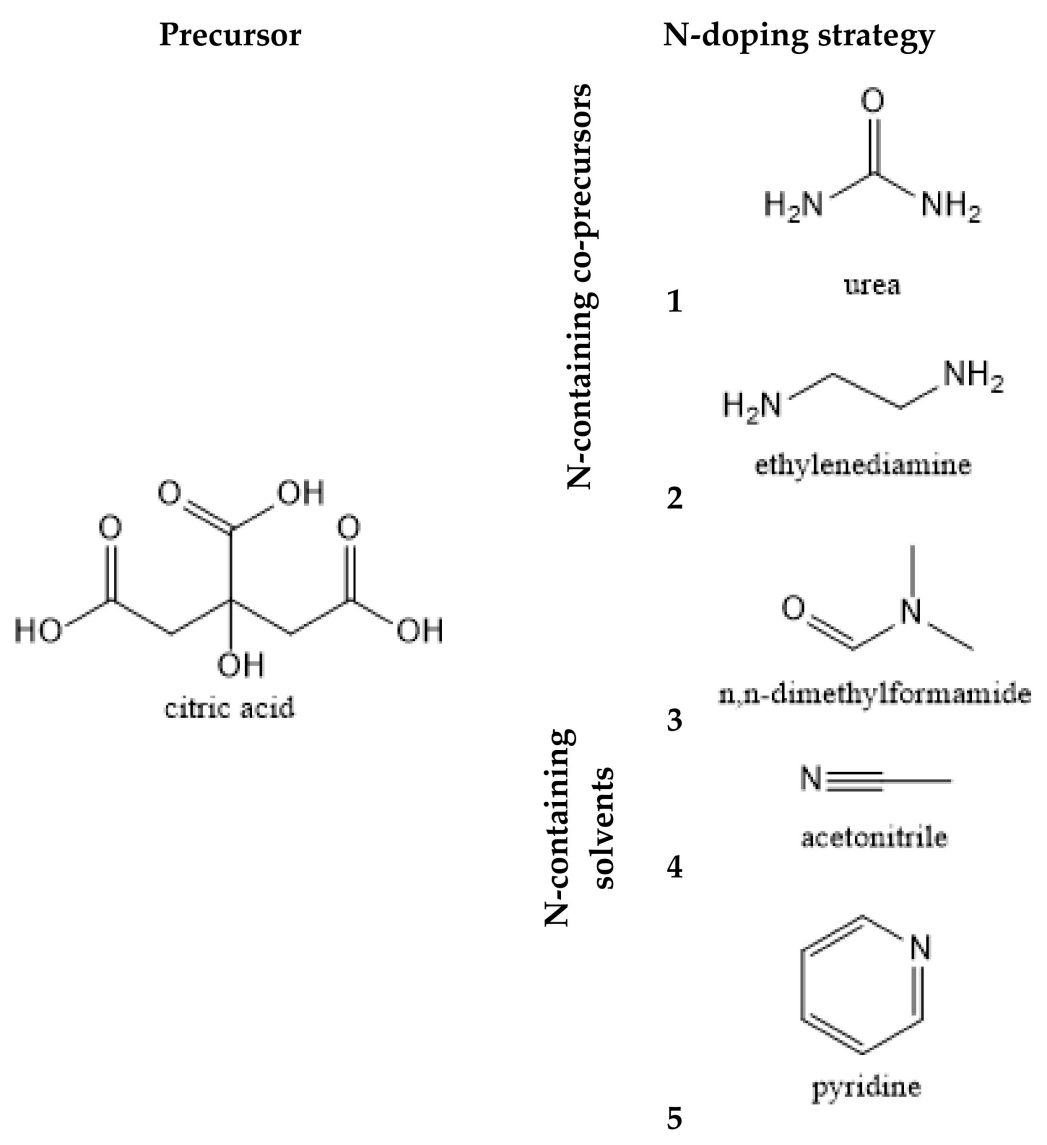
The five synthesis paths used for the preparation of N-doped CDs.

CDs were synthesized by mixing 0.5 g of CA with either an N-containing co-precursor (sample 1: 0.5 g of urea; sample 2: 556 μL of EDA) in 5mL of deionized water, or directly in 5 mL of a N-containing solvent instead of water (sample 3: DMF; 4: acetonitrile; 5: pyridine), as presented in Scheme 1. Each solution was placed in glass beaker and irradiated by microwave treatment for 10 min, by using an Electronia domestic microwave (model P70B17L-DE, with 700 Watts power consumption). The synthesized CDs were subsequently suspended in water, while discarding unsuspended and insoluble aggregates. The solutions were then purified by centrifugation (6000 rpm for 20 min) to eliminate suspended impurities. The samples were subsequently redispersed in 10 mL deionized water and further purified by dialysis (Float-A-Lyzer® G2 Dialysis Device, MWCO: 1 kDa) for 48 h. All purified samples were dried by evaporating the water of the sample by heating to 80 °C. The synthesis yield (weight-by-weight) was calculated considering the amount of final powder and the amount of initial precursors used. 

### 2.2. Characterization of CDs

Zeta potential measurements were performed using a particle analyzer Anton Paar Litesizer™ 500. X-ray photoelectron spectroscopy (XPS) spectra were obtained with a Fi Kratos Axis Ultra HAS-VISION, using monochromatic Al-Kα radiation (15 kV, 90 W). Survey and high-resolution spectra for C 1s, N 1s, and O 1s were acquired at 80 and 40 eV, respectively. Spectra were analyzed and quantified using CasaXPS software employing sensitivity factors supplied by the manufacturer. Analysis included the subtraction of a linear background and charge referencing to the adventitious carbon signal at 285 eV. Spectra were fitted with a series of 70% Gaussian/30% Lorentzian (GL30) line shapes with a width constrained to 0.5–12.5 eV. UV-Vis spectra were obtained with a VWR UV-3100PC Spectrophotometer, using quartz cells. Fluorescence spectra were obtained in standard 10 mm fluorescence quartz cells and collected in a HORIBA Jobin Yvon Fluoromax-4 spectrofluorimeter.

### 2.3. Calculation of Fluorescence Quantum Yield

The fluorescence quantum yield (QY_FL_) was calculated by comparing the integrated luminescence intensities and absorbance values of the synthesized CDs with those of a reference (quinine sulfate or fluorescein depending on the emission wavelength of the sample), with the following Equation:

(1) $$QY_{FL}^{Sample}=\;QY_{FL}^{Reference}\;\times \;\frac{Grad_{Sample}}{Grad_{Reference}}\;\times \;\frac{\eta_{Sample}^{2}}{\eta_{Reference}^{2}},$$

where *Grad* is the gradient from the plot of integrated fluorescence intensity versus absorbance and *η* the refractive index. Quinine sulfate and fluorescein were chosen as reference fluorophores of known quantum yield (QY_FL_ = 0.58 at 350 nm, and 0.95 at 496 nm of excitation wavelength respectively) [42,43,44]. In that case, the fluorescence spectra were measured with a HORIBA Jobin Yvon Fluoromax-4 spectrofluorimeter, and absorbance measurements were made with a UNICAM Helios Gamma, using the same standard 10 mm fluorescence quartz cells.

### 2.4. Scope and System Boundaries 

The aim of the present cradle-to-gate study is to assess the potential environmental impacts of different routes for the bottom-up synthesis of N-doped CDs, which is focused on the laboratory-scale manufacturing stage of the target nanomaterials and considers the direct emissions from CDs’ production and indirect impacts associated with upstream resource extraction and energy generation. This work uses five microwave-assisted synthetic strategies. CA was always used as a carbon source, while a nitrogen-source was provided either by a co-precursor or by the solvent. The environmental impacts were analyzed and compared in a first stage using a weight-based functional unit of 1 kg of CDs. This was made because the selection of a weight- or volume-based functional unit makes sense to compare the production of an equivalent amount of nanomaterial [39,45]. However, these impacts were normalized by the QY_FL_ of the different CDs, in a later stage. Such approach is needed because weight-/volume-based functional units do not consider functional benefits for which they were engineered for, and so, they might not account for the possibility that a more resource-intensive synthesis may be justified later in the use stage (given the improved functionality). The QY_FL_ was chosen as the normalization factor because, while CDs have many different applications, a high QY_FL_ is generally a requested/desired property in most, if not all, of these applications.

### 2.5. Life Cycle Inventory Data 

The assessment of the environmental impacts associated with the bottom-up fabrication of CDs was based on inventory data from laboratory-scale synthesis procedures found in the Ecoinvent® 3.5 database. The synthesis procedure consists of the microwave-based synthesis of the solution for 10 min, followed by centrifugation and purification steps. Therefore, the foreground system considers the industrial production of chemicals used as raw materials for the fabrication of the CDs and the consumption of electricity used for the synthesis process, centrifugation and purification steps. The different processes and chemicals included in this study were modelled with the following data present in the Ecoinvent® 3.5 database (GLO standing for global, RER for regional market for Europe, and PT for Portugal):

- Citric acid {GLO} | market;
- Urea, as N {GLO} | market;
- Ethylenediamine {RER} | market;
- N,N-dimethylformamide {GLO} | market;
- Acetonitrile {GLO} | market;
- Pyridine {GLO} | market;
- Water, deionized, from tap water, at user {Europe without Switzerland} market;
- Electricity, medium voltage {PT} | market;
- Chemical waste, unspecified.

The amounts of chemicals used are described above in the “2.1. Synthesis of Carbon Dots” section, and have been rescaled to the amount needed for the production of 1 kg of CDs. The dataset used for electricity describes the available electricity data available on the medium voltage level in Portugal for the year 2014, as described in the Ecoinvent® 3.5 database. As referred above, the electricity considered combines the electricity required for using the domestic microwave (microwave-assisted synthesis), the centrifugation and purification (dialysis). Microwave-assisted synthesis was made in an Electronia domestic microwave (model P70B17L-DE) with a power consumption of 700 W. Centrifugation was made with a power consumption of 180 W. Stirring of the solution during dialysis was made using a Jenway model 1000 stirrer, with 50 W maximum power consumption. The amounts of electricity consumed for each process are 0.12 kWh for a microwave-assisted synthesis with irradiation duration of 10 min, 0.06 kWh for centrifugation at 6000 rpm for 20 min, and 24 kWh for the stirring of CDs’ solution for 48 h. All processes combined resulted in an electricity consumption of around 24.2 kWh for each synthesis.

### 2.6. Environmental Impact Assessment

The present LCA study is based on a cradle-to-gate approach, from the production of precursor materials to the fabrication of CDs. Environmental impacts were modeled using the ReCiPe 2016 V1.03 endpoint method, Hierarchist version [46]. The impact potentials evaluated according to the ReCiPe method were: Global warming–human health (GW–HH), global warming–terrestrial ecosystems (GW–TE), global warming–freshwater ecosystems (GW–FE), stratospheric ozone depletion (SO), ionization radiation (IR), ozone formation–human health (OF–HH), fine particulate matter formation (FPM), ozone formation–terrestrial ecosystems (OF–TE), terrestrial acidification (TA), freshwater eutrophication (FE), marine eutrophication (ME), terrestrial ecotoxicity (TE), freshwater ecotoxicity (TET), marine ecotoxicity (MET), human carcinogenic toxicity (HC), human non-carcinogenic toxicity (HNC), land use (LU), mineral resource scarcity (MR), fossil resource scarcity (FR), water consumption–human health (WC–HH), water consumption–terrestrial ecosystem (WC–TE) and water consumption–aquatic ecosystems (WC–AE). For simplification, these categories are sometimes presented in three distinct groups: human health, ecosystems, and resources. The LCA study was performed using SimaPro 9.0.0.48 software.

## 3. Results and Discussion

### 3.1. Characterization of Carbon Dots

Synthesis (or reaction) yields (mass by mass, in %) were obtained by measuring the weight of the final amount of powder obtained relative to the initial amount of precursor(s) powder (Table 1). The synthesis yields for the reactions using N-containing co-precursors in water presented relatively good results (close to 8% for CA + urea; and 7% for CA + EDA), as well as the CDs prepared with CA in pyridine solvent (7.3%). On the other hand, the samples prepared with DMF or pyridine solvents presented lower yields (4.4% for CA in DMF and 0.4% for CA in pyridine). The reaction yield using pyridine was particularly low, indicating that this solvent is not suitable for the microwave-assisted synthesis of CDs. Zeta potential measurements showed that CDs should be present in neutral state, as the values are approaching zero mV. Nevertheless, CDs prepared in acetonitrile and pyridine presented small positive and negative (respectively) Zeta potential values.

An XPS analysis was made to understand the surface composition of the five CDs samples. More specifically, the XPS atomic composition (in atomic weight percentage, at.%) of each sample was determined (Table 1). It revealed that all types of samples (both synthetized in water and in N-containing solvents) are composed mostly by C (~60–75 at.%) and O (~16–36 at.%) elements, with different amounts of N (~0.6–15 at.%). Given the objective of this work, it is interesting to see that the use of urea and EDA as precursors were the most effective in doping the surface of the CDs with N heteroatoms (~15 at.%), while microwave-assisted solvothermal strategies were not. In fact, only the synthesis performed in DMF resulted in non-negligible N-doping (~7 at.%), while the N-doping with acetonitrile and pyridine led to negligible results (0.6 and 1.6 at.%, respectively). Thus, in terms of the efficiency of N-doping, the use of organic small-molecules as precursors appears to be the most effective strategy. As for the amount of O and C elements, there is no apparent relationship between the use of either small organic molecules or N-containing solvents on the final composition. 

Detailed scans for the internal levels of C 1s, O 1s, and N 1s were subsequently made. Data processing by deconvolution and peak fitting was done using CasaXPS software, thus providing information on possible chemical groups and their respective contribution (Figures S1–S3 and Tables S1–S3). 

C 1s spectra could be split into four peaks for all samples: At binding energies of ~285 eV attributed to C–C/C–H groups [47], at ~286 eV attributed to amide carbon C–N groups [48,49,50,51], at ~287 eV attributed to ether or alcohol C–O groups [48,49,50,51,52] and at ~288 eV attributed to carbonyl C=O or amide carbon C=N groups [50,51,52,53,54]. All CDs seem to have a high contribution from C–C/C–H groups (between 38% and 62%), a moderate contribution from C–N groups (between 15% and 37%), and a small contribution from C–O groups (between 8% and 11%), although inexistent or undetected contribution in sample 2 (CA-EDA). The contribution of C=O/C=N groups differs for all samples, being quite important for samples 1 (CA-urea) and 4 (CA-acetonitrile) with 36 and 32% respectively, moderate in sample 2 with 23% and relatively small in CDs 3 (CA-DMF) with only 12%. CDs 5 (CA-pyridine) presents a different peak deconvolution around 289 eV as it could be split into two small peaks, with contributions attributed to C=O/C=N at 288.5 eV and to carboxylate (O-C=O) or amide carbon (N-C=O) at 289.3 eV [48,55,56,57,58], with 6% and 8% respectively thus giving an overall contribution of 14% for the peak at 289 eV. The presence of π-conjugated groups (C=O, C=N, O-C=O and N-C=O) has a potential impact on the photoluminescence efficiency of CDs [59,60,61,62]. The C 1s analysis indicates a moderate degree of π-conjugation at the surface of CDs for samples 1, 2 and 4 with 36%, 23% and 32% respectively, due to C=O/C=N groups, and a smaller π-conjugation for CDs 3 and 5 with only 12% and 14% respectively.

O 1s could be split into just two peaks: at binding energy of ~532 eV (C=O or N-C=O groups) and ~533 eV (C–O groups) [48,51,52,53,58]. The five CDs show a predominant contribution from C=O groups (~55–84%), and moderate contribution from C–O groups (~16–45%). However, this predominance of C=O groups is higher for samples 1 and 2 (synthesized using small organic small-molecules as precursors) with more than 80% contribution.

N 1s spectra can be split into several peaks depending on the samples, with two peaks being present in all CDs at binding energies ~400 eV and ~401.5 eV, that can be attributed to pyrrolic N (also called C_2_-N-H) and graphitic N (also called N-C_3_) respectively [51,54,63]. The two first samples present dominant contributions from pyrrolic N group (~89%) and small contribution from graphitic N group (~11%). Additionally, another peak is found in sample 3 at ~402 eV, which can be attributed to N-O bond [57,64]. Due to the quite small amount of N-doping (0.6 and 1.6%), the N 1s spectra of samples 4 and 5 were not analyzed.

### 3.2. Optical Properties of Carbon Dots

The optical properties of the five CDs have been characterized by UV-Vis spectroscopy and fluorescence spectroscopy (Table 2). 

**Figure 1:**
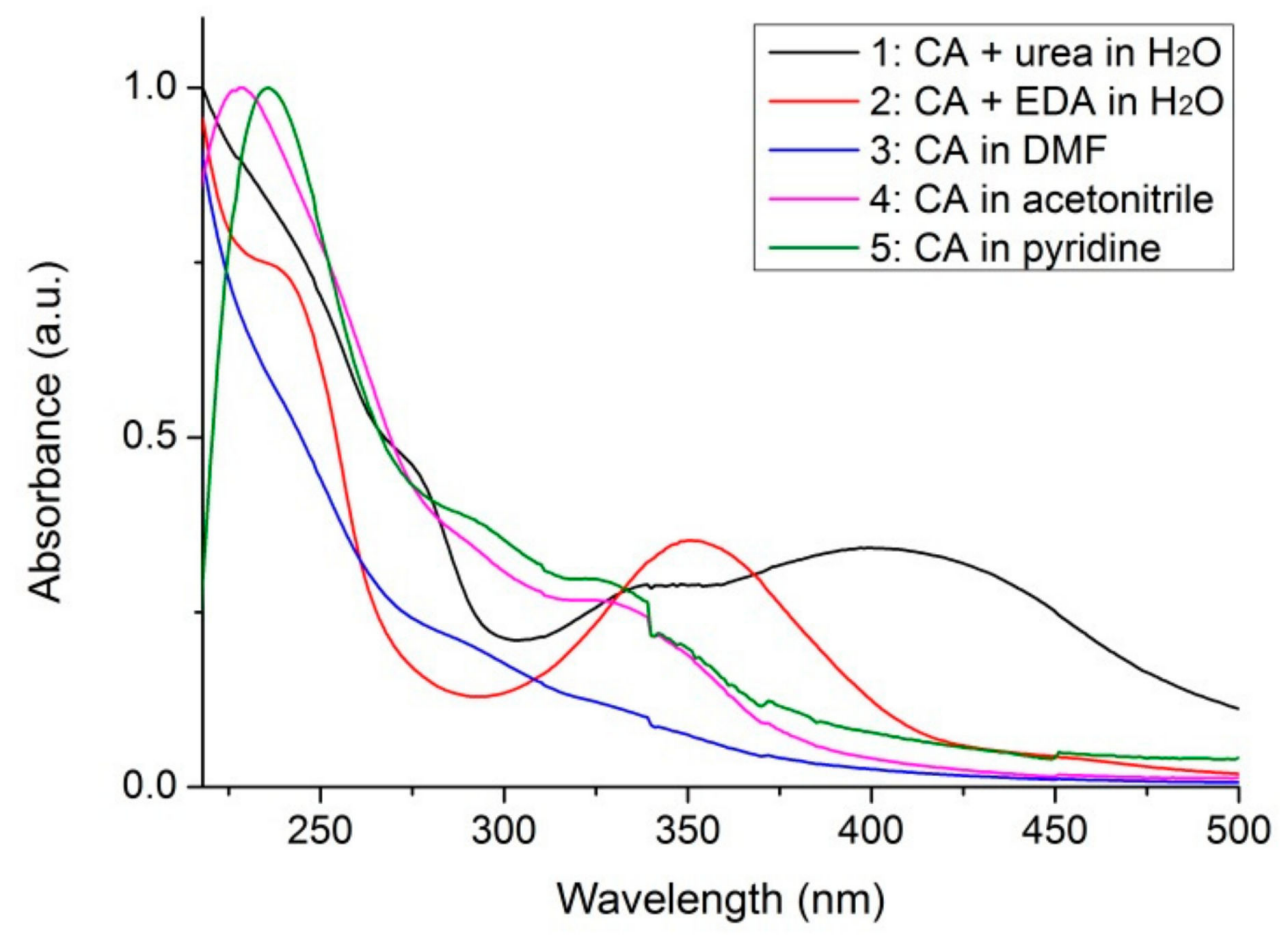
Absorption spectra of the five CDs. Data were normalized.

The absorption spectra of the five CDs can be found in Figure 1. All spectra exhibit band-to-band core transitions below 300 nm [65], with sample 2 presenting a shoulder around 245 nm that can be due to the π-π* transition of the C=C bond [26,50]. Sample 1 presents two shoulders around 245 and 275 nm. Above 300 nm, samples 1 and 2 present an absorption region around 340 nm can be assigned to n-π* transitions at the edge of the carbon lattice [54,66]. Additionally, sample 1 presents an absorption band at 410 nm. Samples 3, 4 and 5 present all the same absorption features below 300 nm, with a small shoulder around 340 nm, followed by an absorption decreasing up to 500 nm. 

**Figure 2:**
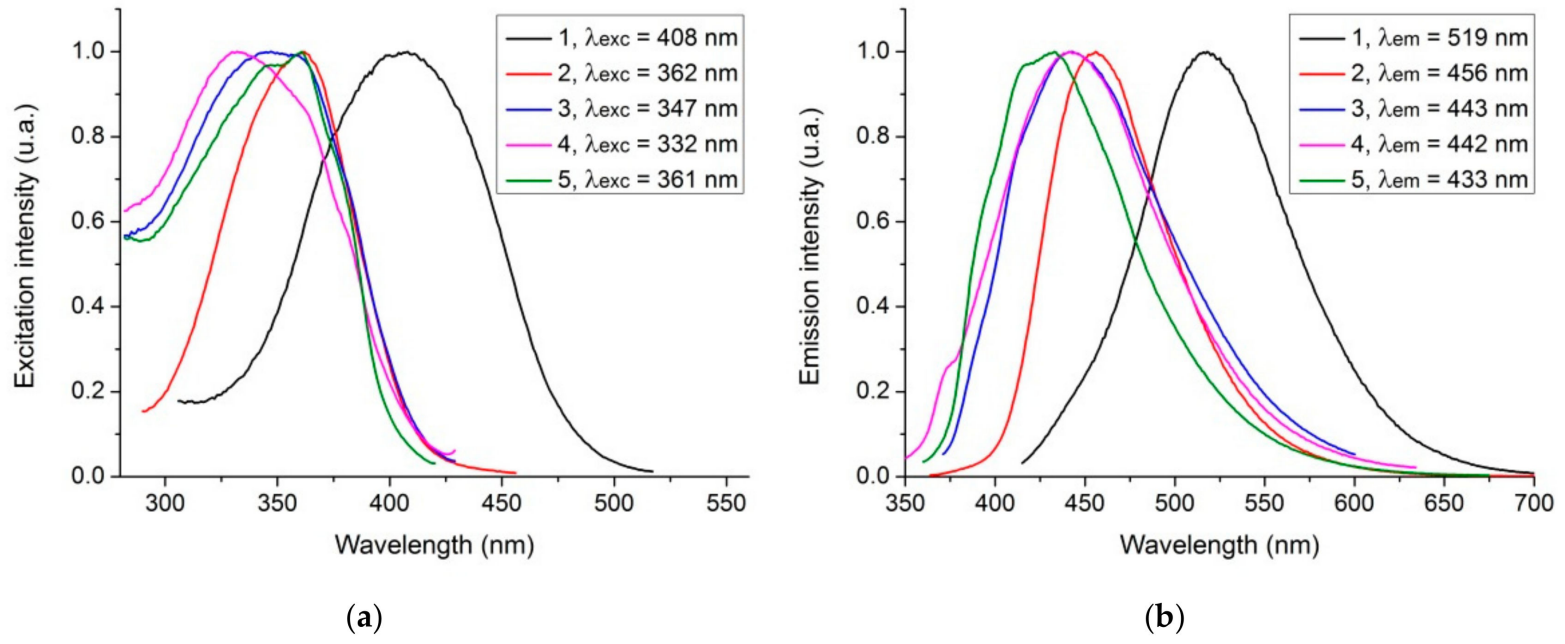
Fluorescence (**a**) Excitation spectra and (**b**) Emission spectra of the five CDs. Data were normalized to their maximum.

The fluorescence excitation and emission spectra and wavelength maxima of the five samples can be found in Figure 2 and Table 2. Apart from sample 1 (CA-urea), all four CDs (2, 3, 4 and 5) present similar fluorescence characteristics with comparable excitation maxima: 360 nm for samples 2 (CA-EDA) and 5 (CA-pyridine), 350 nm for sample 3 (CA-DMF), and 330 nm for sample 4 (CA-acetonitrile). Similarly, they generate blue emission with fluorescence maxima: 460 nm for CDs 2, 440 nm for both CDs 2 and 3, and 430 nm for CDs 5. Sample 1, on the other hand, shows an excitation maximum at 410 nm and a fluorescence maximum at 520 nm. It should also be noted that while the QY_FL_ of samples 2-5 is moderate, the one of sample 1 is quite high (Table 2). Interestingly, there is no apparent correlation between the amount of N-doping (Table 1) and the QY_FL_ (Table 2), contrary to what could be expected from the literature [27,28,29]. However, it should be noted that while N-doping is generally correlated with fluorescence enhancement, it is not really known how N-doping leads to such phenomenon. Thus, our results indicate that the mechanism of fluorescence enhancement via N-doping require further study.

### 3.3. Improvement on the Purification of CA-urea Carbon Dots

The results presented in the previous section indicate that the most attractive synthesis strategy is the one based on the hydrothermal treatment with addition of urea, given that it presented a significantly higher QY_FL_ and a more red-shifted emission, while presenting also one of the highest N-doping efficiency and synthesis yield. However, the fact that the QY_FL_ of sample 1 is so much higher than those of the other four samples, which are similar between them, indicates that we must analyze these results with caution. The same can also be said for the differences between the fluorescence spectra of sample 1 and samples 2–5. In fact, the absorption, fluorescence excitation and emission spectra of sample 1 are in line with the fluorescent impurities generated in a similar synthesis of CDs from CA and urea [17]. 

Given this, we repeated the synthesis of sample 1 but changed the dialysis parameters to ensure a more effective purification. Namely, we prepared a sample 1B that was dialyzed at the same cut-off value (1kDa), but for 96 h instead of 48 h. A sample 1C was also prepared, which was dialyzed at a higher cut-off value (3.5 kDa) for 96 h. The CDs indicated before as sample 1 has been renamed “1A” in this section for comparison purposes.

In fact, the modification of purification protocol led to significant changes (Table 3). In particular, the reaction yield of samples 1B and 1C is significantly lower than for sample 1A, which indicates that the previous purification protocol was not sufficient to remove impurities from this synthesis. Moreover, the new purification protocols also led to a significant reduction of the QY_FL_ (Table 3) to values comparable to those obtained for samples 2–5.

**Figure 3:**
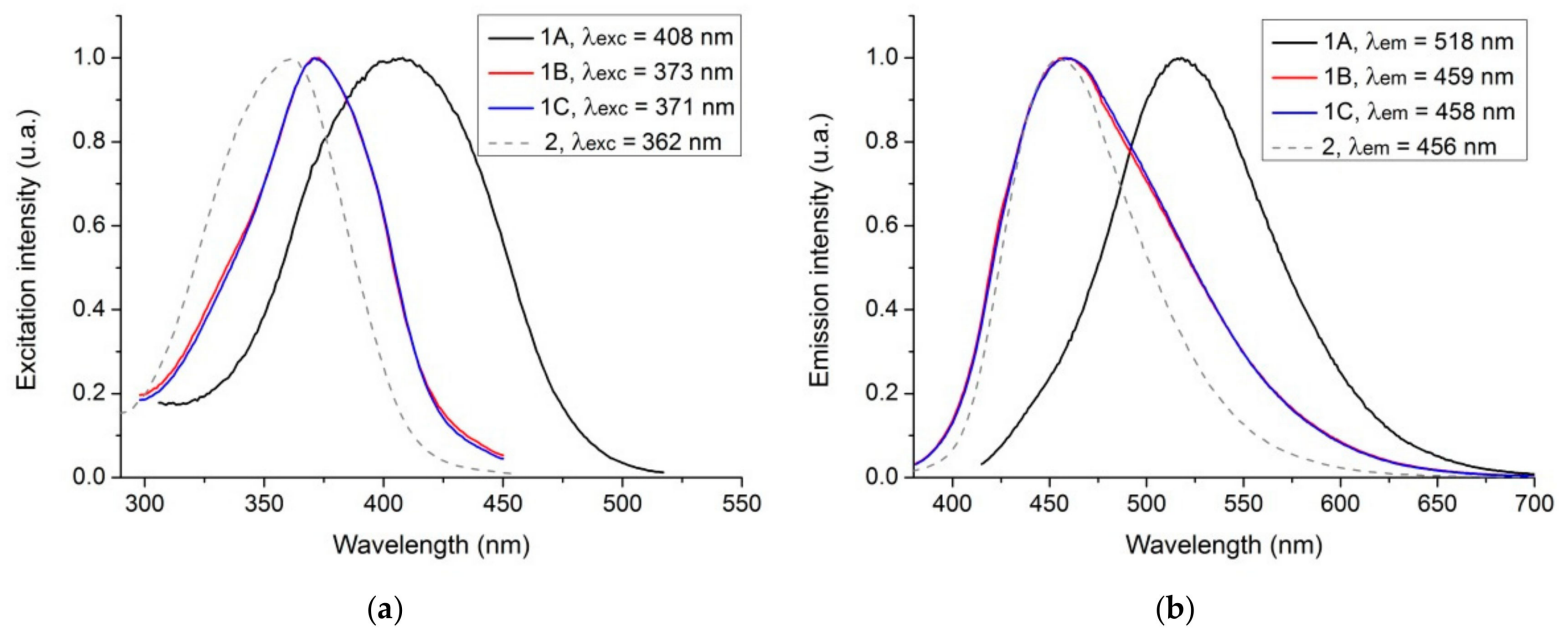
Fluorescence (**a**) excitation spectra and (**b**) emission spectra of carbon dots 1A, 1B, 1C, and 2. Data were normalized.

The fluorescence excitation and emission spectra are also affected (Figure 3), with samples 1B and 1C presenting a relevant blue-shift compared to sample 1A. It should also be stated that this blue-shift put the fluorescence profile of samples 1B and 1C closer to what is observed in sample 2 (Figure 3).

The absorption spectra for the three CDs (presented in Figure S4), all show a similar profile, with a high absorption before 300 nm, and two broad peaks at 350 nm and around 400 nm. CDs 1A presents an extra shoulder at 275 nm, and a small red shift for the peak at 400 nm (as compared to 380 nm for CDs 1B and 1C), that could be explained by the presence of impurities. 

The surface composition of these CDs was also analyzed by XPS. All three samples display a similar composition, containing mostly carbon (~61%–65%), oxygen (~21–24%) and nitrogen (~14%–16%) elements. Detailed spectra for CDs 1A and 1B are shown in the Supporting Information (Figure S5). As before, C 1s spectra could be split into four peaks for all samples: At binding energies of ~285 eV (C–C/C–H groups), ~286 eV (C–N groups), ~287 eV (C–O groups), and ~288 eV (C=O/C=N). All CDs samples present a very similar composition, with predominant contribution from C–C/C–H groups (~37%–49%), followed by C=O/C=N groups (~25–36%), then C–N groups (~15%–18%) and small contribution of C–O groups (~9%–12%). O 1s could be split into just two peaks: At binding energy of ~532 eV (C=O groups) and ~533 eV (C–O groups). The three CDs present identical composition: Predominant contribution by C–O groups (~84%–86%), with moderate contribution from C=O groups (~14%–16%). N 1s could also be split into two peaks: At binding energies of ~400 eV (pyrrolic N) and ~402 eV (graphitic N). All samples present dominant contributions from pyrrolic N groups (~88%–91%), and small contributions from graphitic N groups (~9%–12%). 

After characterization of CA-urea CDs with a higher degree of purification, it can be concluded that the properties of sample 1A are not representing only those of CDs but also of fluorescent impurities present in solution. As CDs 1B and 1C present similar structure and optical properties, the sample 1B was chosen for study in the next section as its purification parameters are closest to other samples studied: It was purified for twice the amount of time (96 h instead of the previous 48 h) while keeping the same molecular weight cut-off (MWCO at 1 kDa).

### 3.4. Life Cycle Assessment Study

In this section, the five different synthesis routes were first evaluated individually to analyze their impacts contributions in each category (as described in Section 2.6 “Environmental Impact Assessment”) using a weight-based functional unit for the production of 1 kg of CDs. Then, these synthesis processes were compared to evaluate their relative environmental impacts. Finally the same comparison was done using a function-based functional unit using the fluorescence quantum yield (QY_FL_) of each CDs solution produced.

As it was discussed earlier in Section 3.3 “Improvement on the Purification of CA-Urea Carbon Dots”, sample 1B was chosen to replace 1A in the first synthesis route. The modified dialysis parameters were incorporated in the calculations, by increasing the amount of electricity used by the stirring plate and amount of water used for dialysis (as shown in Table S4). Thus, the environmental impacts of samples 1B, 2, 3, 4, and 5 are presented in Figure S6A–E, respectively. CDs 1B (Figure S6A) present its highest contribution to environmental impacts from the use of CA in almost all categories, except in terrestrial ecotoxicity (TE) and fossil resource scarcity (FR) categories, where the major contribution is due to urea. Electricity and water consumption have small to negligible impact as compared to chemicals, since the microwave-assisted synthesis is not demanding in those parameters. Similarly, CDs 2 (Figure S6B) contributions are mainly due to CA, except in categories of ionization radiation (IR), marine eutrophication (ME), and fossil resource scarcity (FR), where the use of EDA plays a major role. Again, electricity and water consumption have small contributions, but slightly higher than in the case of sample 1B. For the next three synthesis (CDs 3, 4, and 5) (Figure S6C–E, respectively), the main source of impact comes from the N-containing solvent (DMF, acetonitrile and pyridine respectively), while electricity and water contributions are negligible as compared to the impacts from chemicals. The use of CA has a major contribution in the categories of land use (LU) and water consumption on aquatic ecosystems (WC-AE) for the three CDs, with an additional major effect on stratospheric ozone depletion (SO) in the case of sample 4.

**Figure 4:**
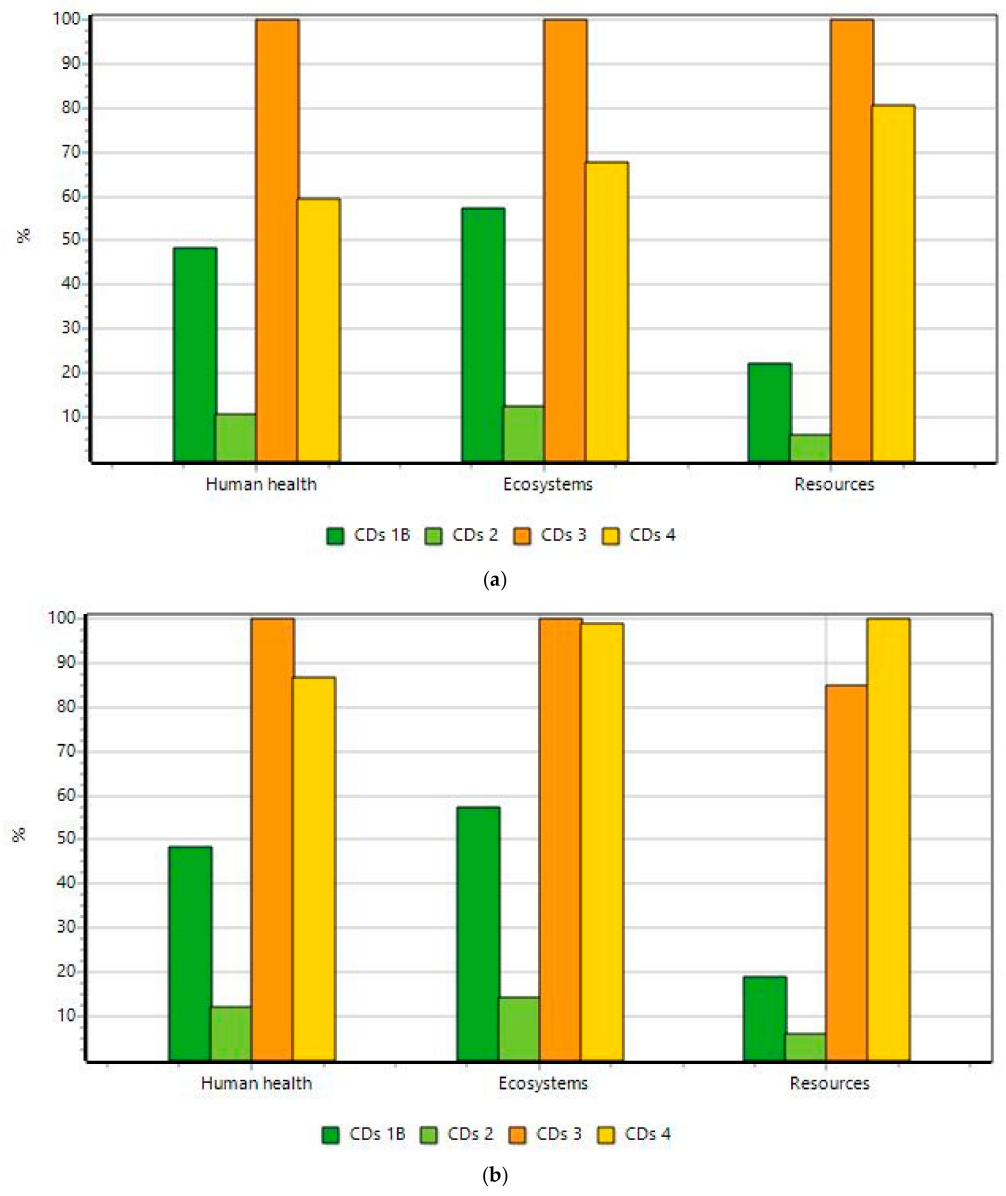
Comparative damage assessment of CDs 1B to 4 (**a**) using weight-based functional unit analysis for 1 kg of CDs produced and (**b**) using QY_FL_–based functional unit analysis.

Sample 5 does not give satisfactory results in reaction yield (only 0.4%), and so, it requires high amounts of chemicals (i.e., 278 kg of CA and 2720 kg of pyridine for the production of 1 kg of CDs which is more than 200 times the amount required for CDs 2, and 12 times the amount for CDs 3) (Table S4). Thus, this sample is excluded from the following comparison of impacts. When comparing the relative impacts of the syntheses, first using a weight-based functional unit (Figure 4a), the best choice in all categories appears to be sample 2 (made from CA and EDA).

Once again, when considering the QY_FL_ of the CDs produced (in Figure 4b), the environmental impacts seem to smaller for sample 2 with respect to other samples. Overall, CDs 2 is clearly the least demanding in resources compared to all other CDs. Thus, the microwave-assisted synthesis of CDs in water solvent using CA and EDA as precursors presents itself as the most interesting for clean production in our study.

## 4. Conclusions

To conclude, the efficiency and environmental impacts of N-doping strategies in the synthesis of CDs were evaluated. Namely, we prepared N-doped CDs from a standard precursor (CA) via two different strategies: (1) Microwave-assisted hydrothermal synthesis with addition of small N-containing organic molecules (urea or EDA); (2) microwave-assisted solvothermal synthesis in different N-containing solvents (DMF, acetonitrile or pyridine). Despite these syntheses producing CDs with similar blue emission, XPS analysis revealed that N-doping was only effective for hydrothermal strategies involving urea and EDA, and DMF-based solvothermal treatment. Moreover, among these latter three strategies, only the ones involving EDA and DMF presented non-negligible reaction yields. An LCA study was subsequently made by considering both the reaction yield and the QY_FL_ as the functional unit. These results showed that the most environmentally sustainable strategy that presented the least environmental impacts, with a significant distance from the other routes, was that of microwave-assisted hydrothermal treatment with addition of EDA.

## Figures and Tables

**Table 1:** Results of the characterization of the five carbon dots (CDs), including reaction yield, zeta potential, and atomic composition (carbon, oxygen and nitrogen, obtained by XPS analysis).

CDs	1: Citric Acid (CA) + Urea in H_2_O	2: CA + Ethylenediamine (EDA) in H_2_O	3: CA in *n*,*n*-Dimethylformamide (DMF)	4: CA in Acetonitrile	5: CA in Pyridine
Reaction yield (%)	7.9	7.3	4.4	7.3	0.4
Zeta potential (mV)	−0.6	−0.2	0.6	2.1	−2.4
C 1s (at.%)	60.9	68.5	70.9	63.1	74.8
O 1s (at.%)	23.7	16.5	22.2	36.4	23.6
N 1s (at.%)	15.4	15.0	6.9	0.6	1.6

**Table 2:** Fluorescence quantum yield (QY_FL_, in %), excitation maximum (λexc, in nm) and emission maximum wavelengths (λem, in nm) for the five studied CDs.

CDs	1	2	3	4	5
QY_FL_ (%)	37.4	3.1	3.5	2.4	2.3
λexc (nm)	410	360	350	330	360
λem (nm)	520	460	440	440	430

**Table 3:** Results obtained for carbon dots 1A (1 kDa, 48 h), 1B (1kDa, 96 h) and 1C (3.5 kDa, 96 h).

CDs	1A	1B	1C
Reaction yield (%)	7.9	1.4	1.8
Zeta potential (mV)	−0.6	−1.4	−3.3
C 1s (at.%)	60.9	61.7	65.2
O 1s (at.%)	23.7	22.8	21.0
N 1s (at.%)	15.4	15.6	13.9
QY_FL_ (%)	37.4	3.5	3.3
λexc (nm)	410	370	370
λem (nm)	520	460	460

## Supplementary Materials

The following are available online at https://www.mdpi.com/1996-1944/13/3/504/s1, Figure S1: Detailed XPS spectra of C 1s (carbon dots 1 to 5); Figure S2: Detailed XPS spectra of O 1s (carbon dots 1 to 5); Figure S3: Detailed XPS spectra of N 1s (carbon dots 1 to 5); Figure S4: Absorbance spectra of carbon dots 1A, 1B, and 1C; Figure S5: XPS spectra C 1s (left) and N 1s (right) for carbon dots 1A, 1B, and 1C; Figure S6: Relative environmental impacts of each carbon dots solution with the ReCiPe2016 LCIA for: (A) CDs 1B, (B) CDs 2, (C) CDs 3, (D) CDs 4, and (E) CDs 5; Table S1: Contributions (%) to C 1s peaks after deconvolution (CasaXPS software); Table S2: Contributions (%) to O 1s peaks after deconvolution (CasaXPS software); Table S3: Contributions (%) to N 1s peaks after deconvolution (CasaXPS software); Table S4: Quantity of products required for the production of 1 kg of CDs (SimaPro software); Table S5: Fluorescence quantum yields (QY_FL_) and QY_FL_-based functional units.
