# Supplementary material for: Evaluation of the Environmental Impact and Efficiency of N-Doping Strategies in the Synthesis of Carbon Dots
Source: Materials (Basel). 2020 Jan 21;13(3):504. doi: 10.3390/ma13030504 (PMC7040597; doi:10.3390/ma13030504)
Supplement: Supplementary file 1 [file materials-13-00504-s001.pdf]

materials

MDPI

Article

Evaluation of the Environmental Impact and Efficiency of N-Doping Strategies in the Synthesis of Carbon Dots

Suzanne Christé [1], Joaquim C.G. Esteves da Silva [1,2] and Luís Pinto da Silva [1,2,*]

[1] Chemistry Research Unit (CIQUP), Faculty of Sciences of University of Porto, R. Campo Alegre 697, 4169–007 Porto, Portugal; christe.suzanne@gmail.com (S.C.); jcsilva@fc.up.pt (J.E.d.S.)

[2] LACOMEPHI, GreenUPorto, Department of Geosciences, Environment and Territorial Planning, Faculty of Sciences of University of Porto, R. Campo Alegre 697, 4169–007 Porto, Portugal.

* Correspondence: luis.silva@fc.up.pt.

Received: 03 January 2020; Accepted: 16 January 2020; Published: date

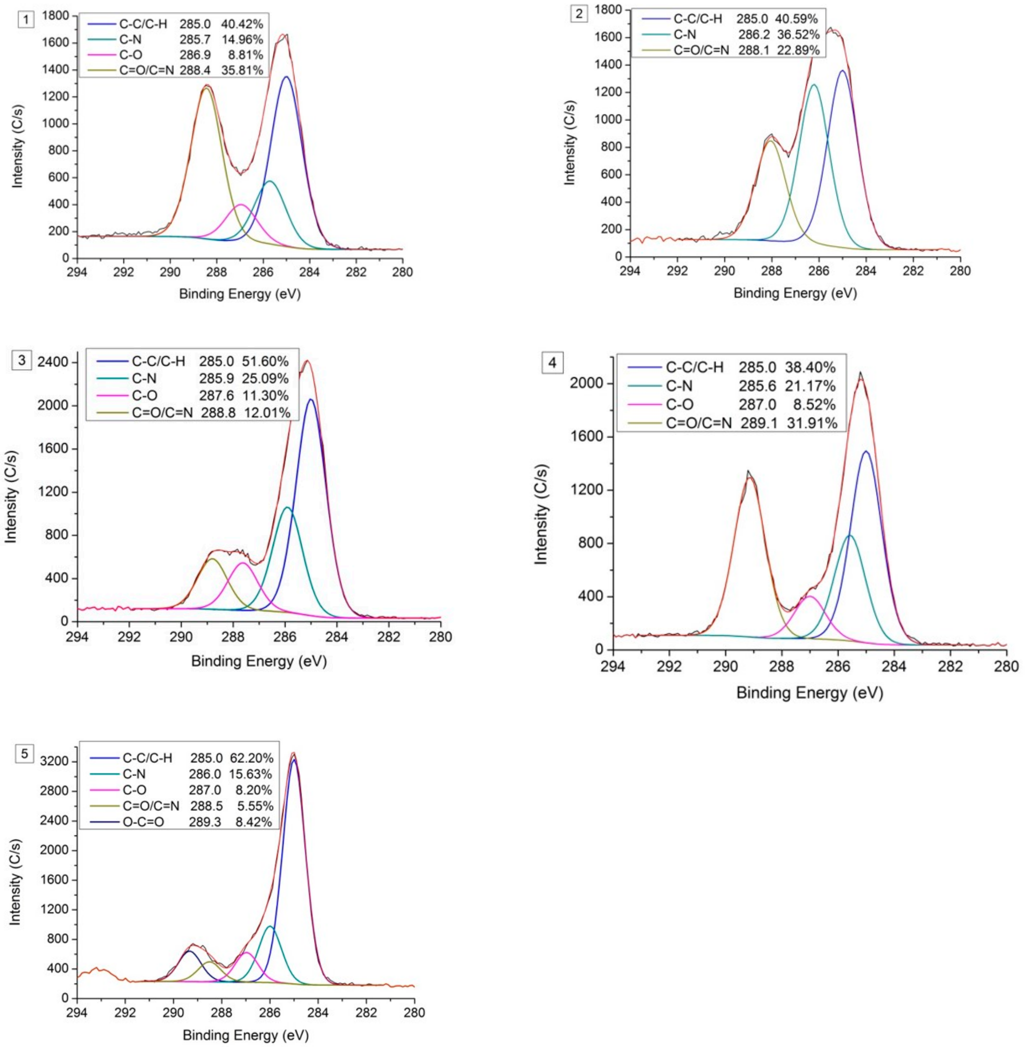

Figure S1. Detailed XPS spectra of C 1s (Carbon dots 1 to 5).

Table S1. Contributions (%) to C 1s peaks after deconvolution (CasaXPS software).

C 1s bond (BE)	1	2	3	4	5
C-C/C-H (~285 eV)	40	41	52	38	62

C-N (~286 eV)	15	37	25	21	16
C-O (~287 eV)	9	-	11	9	8
C=O/C=N (~288 eV)	36	23	12	32	6
O-C=O (~289 eV)	-	-	-	-	8

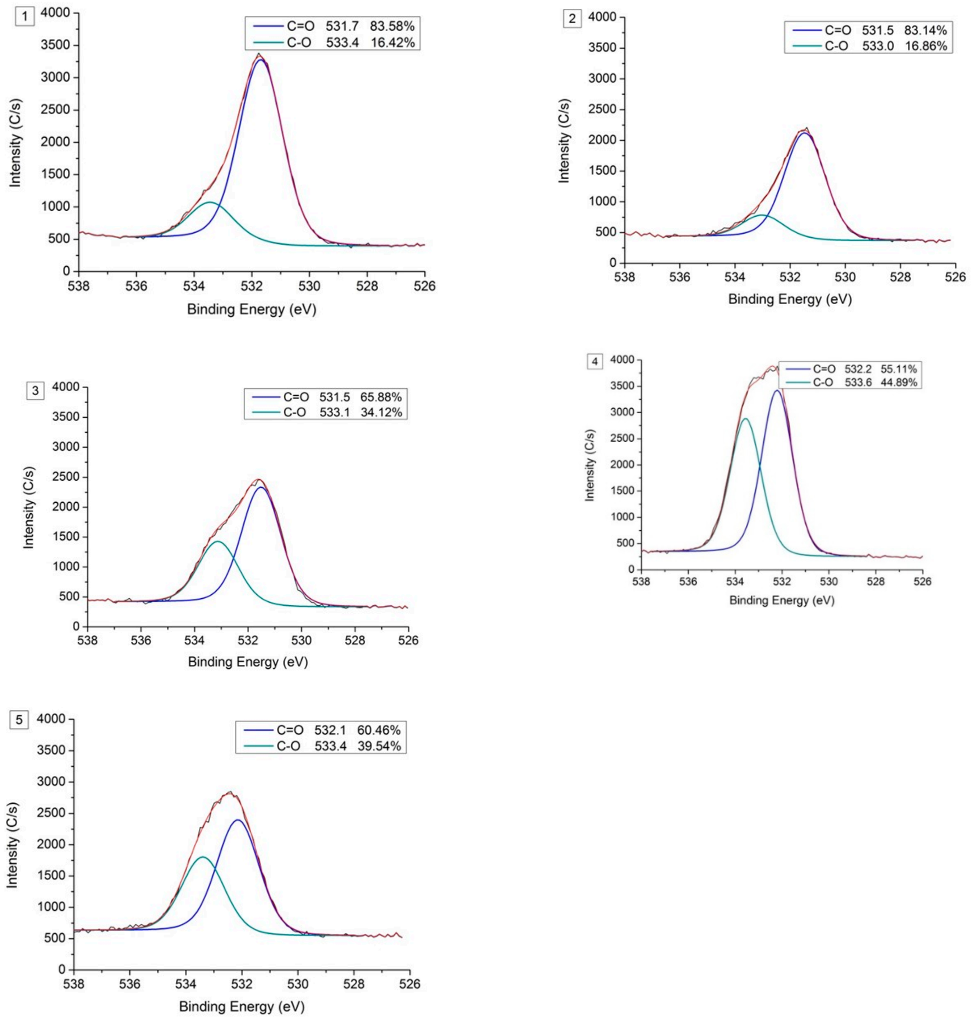

Figure S2. Detailed XPS spectra of O 1s (Carbon dots 1 to 5).

Table S2. Contributions (%) to O 1s peaks after deconvolution (CasaXPS software).

O 1s bond (BE)	1	2	3	4	5
C=O (~532 eV)	84	83	66	55	60
C-O (~533 eV)	16	17	34	45	40

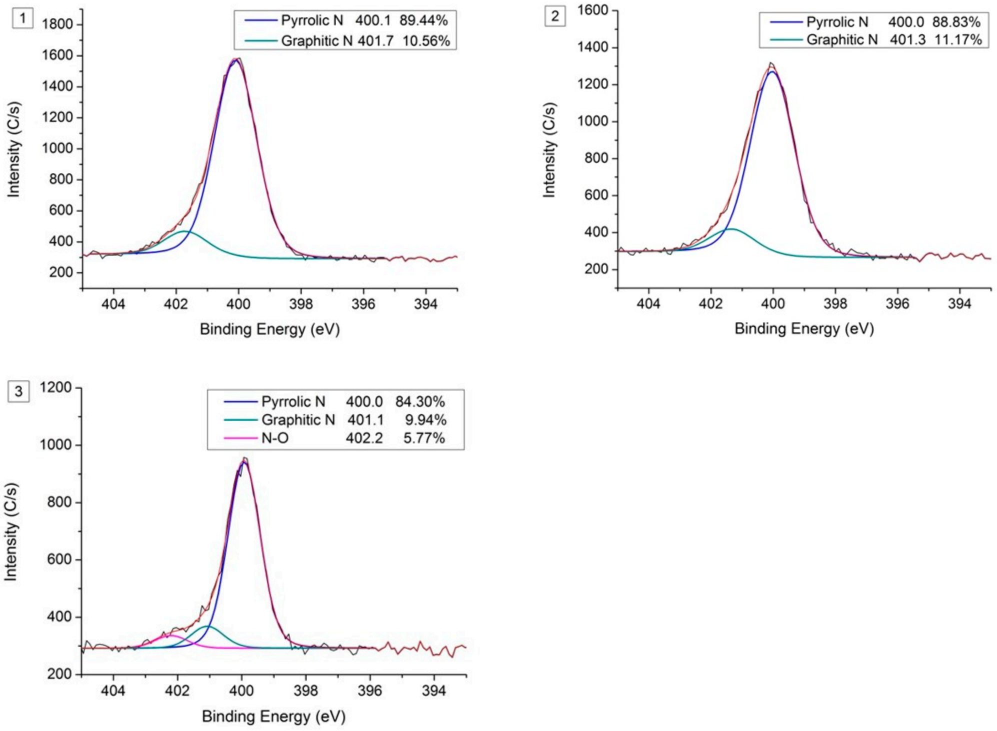

Figure S3. Detailed XPS spectra of N 1s (Carbon dots 1 to 3).

Table S3. Contributions (%) to N 1s peaks after deconvolution (CasaXPS software).

N 1s bond (BE)	1	2	3
Pyrrolic N (~400 eV)	89	89	84
Graphitic N (~401.5 eV)	11	11	10
N-O (~402 eV)	-	-	6

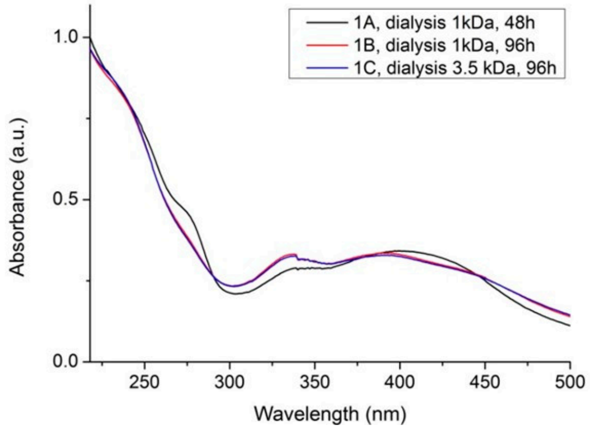

Figure S4. Absorbance spectra of carbon dots 1A, 1B and 1C.

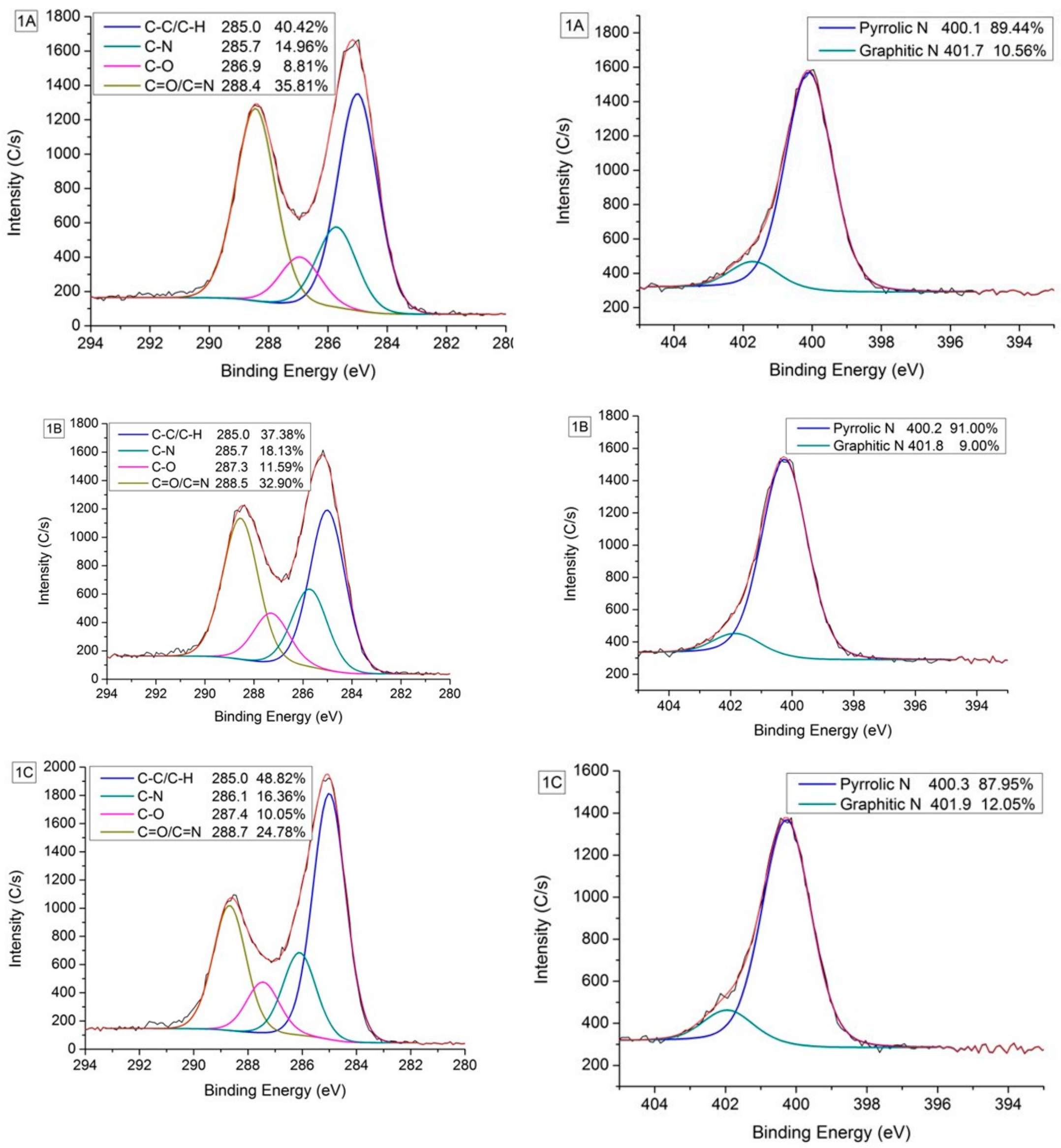

Figure S5. XPS spectra C 1s (left) and N 1s (right) for carbon dots 1A, 1B and 1C.

Table S4. Quantity of products required for the production of 1 kg of CDs (SimaPro software).

Carbon dots	Citric acid (kg)	Nitrogen source (kg)	Deionized water (kg)	Electricity (MJ)
**1B**	35	35	357	174
**2**	7.2	7.2	77.9	87.1
**3**	22.9	217	5.5	87.1
**4**	13.7	108	5.5	87.1
**5**	278	2720	5.5	87.1

Table S5. Fluorescence quantum yields (QY_{FL}) and QY_{FL}-based functional units.

Carbon dots	QY_{FL} (%)	QY_{FL}-based functional unit (using 1B as reference)
**1B**	3.5	1.0

**2**	3.1	1.1
**3**	3.5	1.0
**4**	2.4	1.5
**5**	2.3	1.5

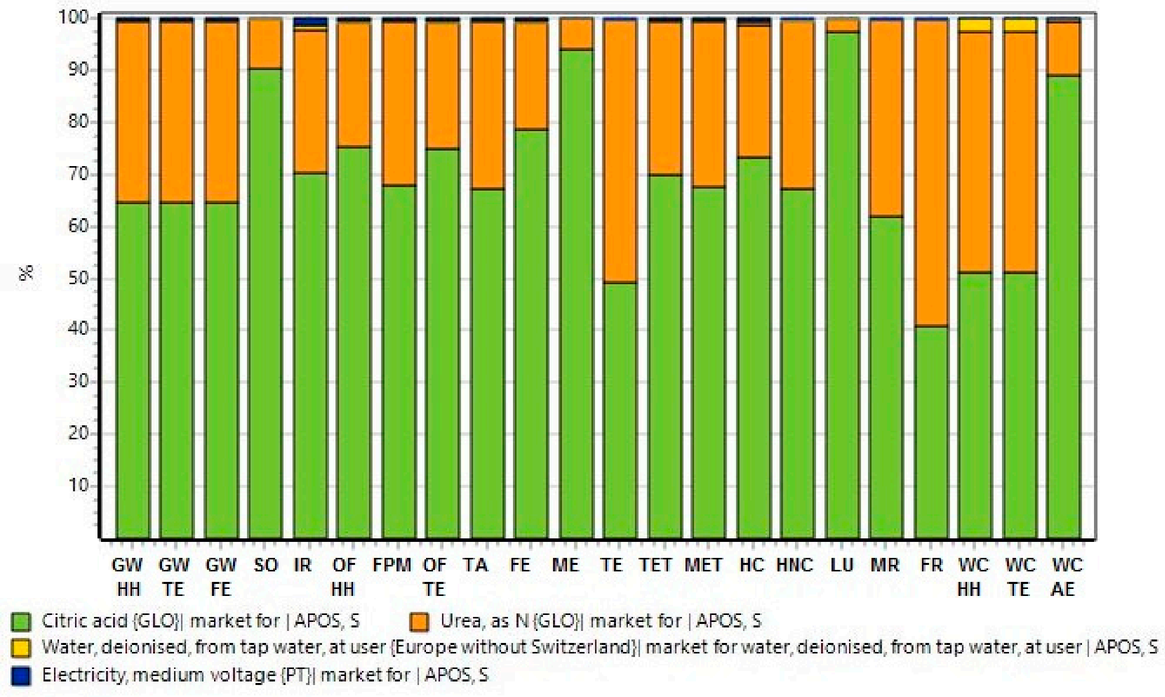

(**A**)

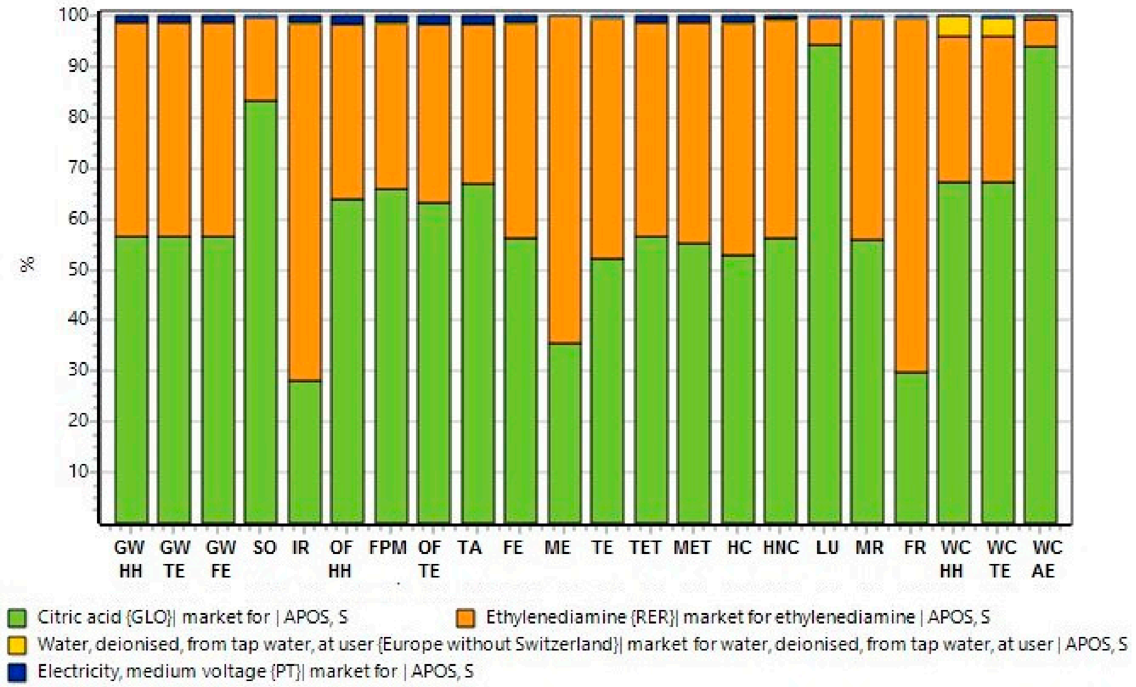

(**B**)

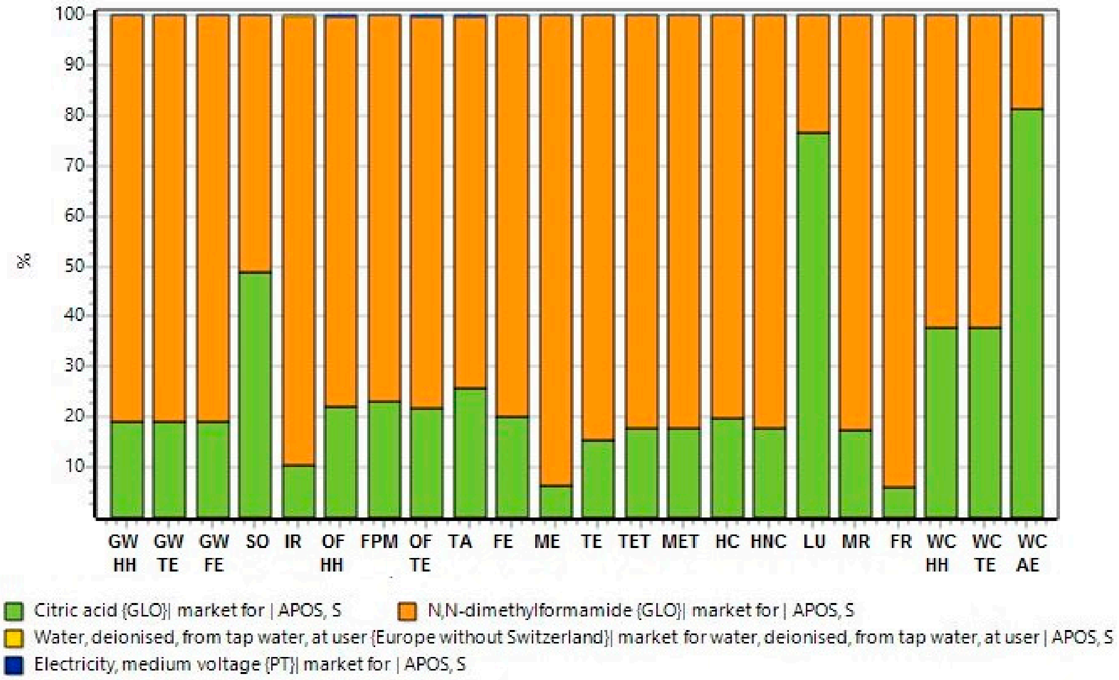

%
100
90
80
70
60
50
40
30
20
10
GW HH
GW TE
GW FE
SO
IR
OF HH
FPM
OF TE
TA
FE
ME
TE
TET
MET
HC
HNC
LU
MR
FR
WC HH
WC TE
WC AE
Citric acid {GLO}| market for | APOS, S
N,N-dimethylformamide {GLO}| market for | APOS, S
Water, deionised, from tap water, at user {Europe without Switzerland}| market for water, deionised, from tap water, at user | APOS, S
Electricity, medium voltage {PT}| market for | APOS, S

(**C**)

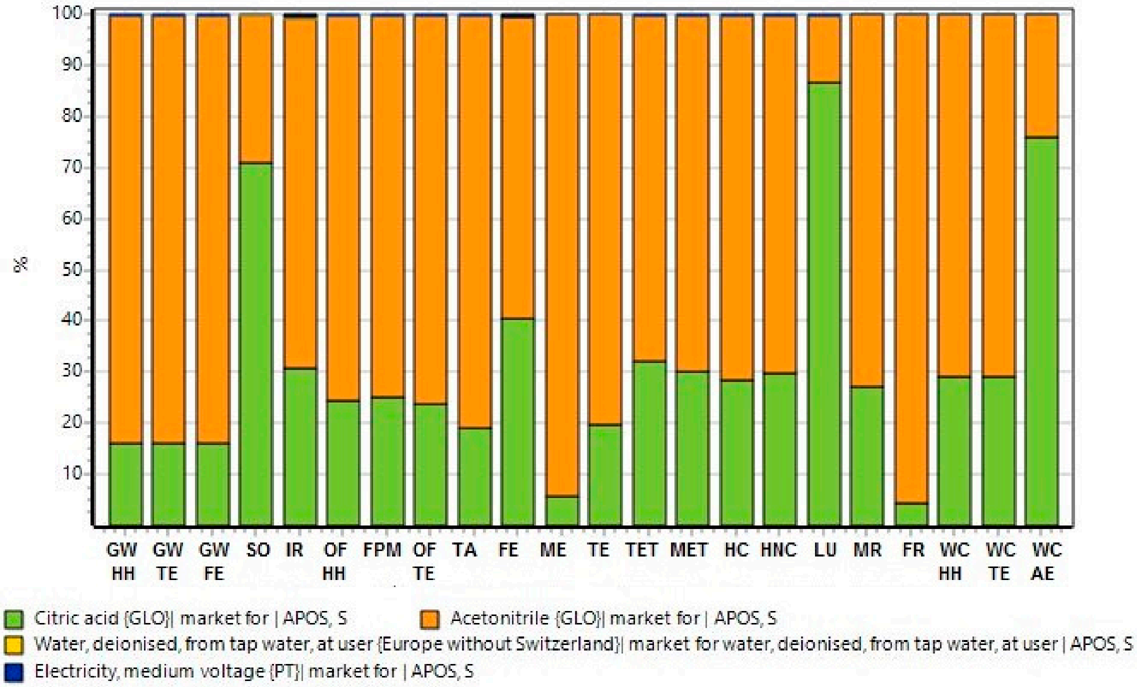

%
100
90
80
70
60
50
40
30
20
10
GW HH
GW TE
GW FE
SO
IR
OF HH
FPM
OF TE
TA
FE
ME
TE
TET
MET
HC
HNC
LU
MR
FR
WC HH
WC TE
WC AE
Citric acid {GLO}| market for | APOS, S
Acetonitrile {GLO}| market for | APOS, S
Water, deionised, from tap water, at user {Europe without Switzerland}| market for water, deionised, from tap water, at user | APOS, S
Electricity, medium voltage {PT}| market for | APOS, S

(**D**)

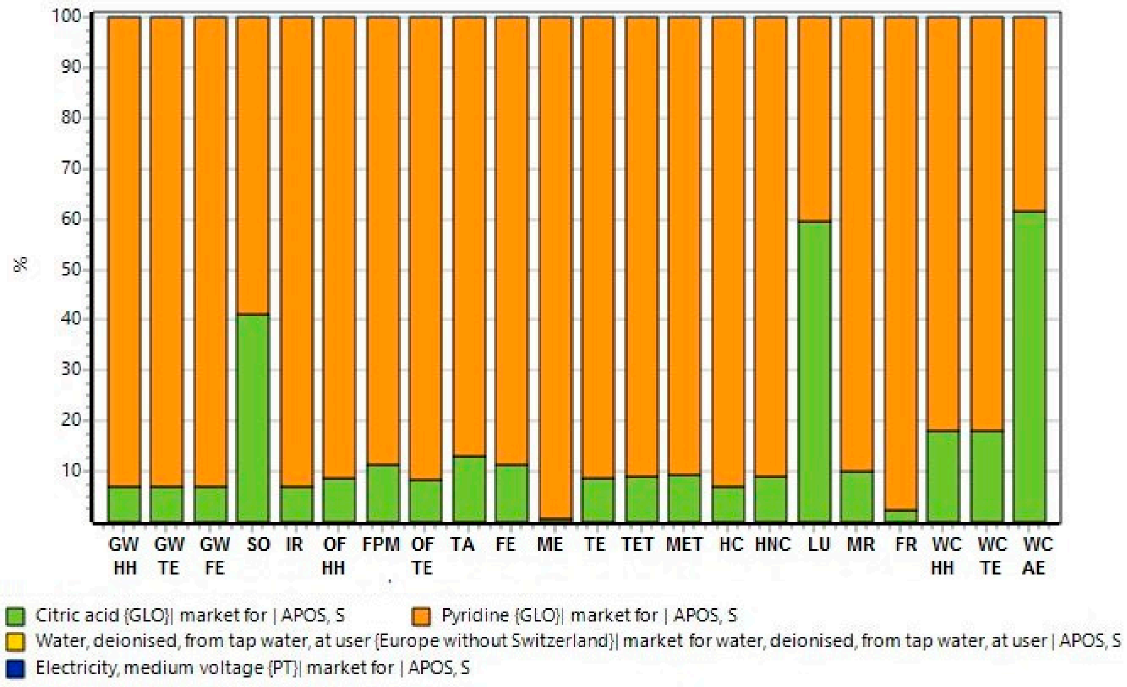

(**E**)

Figure S6: Relative environmental impacts of each carbon dots solution with the ReCiPe2016 LCIA for: (**A**) CDs 1B, (**B**) CDs 2, (**C**) CDs 3, (**D**) CDs 4 and (**E**) CDs 5.
